# The Head Start Tobacco Cessation Initiative: Using Systems Change to Support Staff Identification and Intervention for Tobacco Use in Low-Income Families

**DOI:** 10.1007/s10900-014-9827-9

**Published:** 2014-02-17

**Authors:** Sarah Moody-Thomas, Michael Sparks, Laura Hamasaka, Sarah Ross-Viles, Amber Bullock

**Affiliations:** 1 School of Public Health, Louisiana State University Health Sciences Center, New Orleans, LA USA; 2 M & N Sparks, Inc., Kihei, HI USA; 3 Public Health – Seattle and King County, Seattle, WA USA; 4 Legacy, 1724 Massachusetts Ave NW, Washington, DC 20036 USA

**Keywords:** Tobacco, Smoking, Head Start, Early childhood education, Secondhand smoke exposure, Systems change

## Abstract

Tobacco use continues to be the leading cause of preventable illness and death in the United States. Remarkably, more than nine million preschool-aged children are exposed to secondhand smoke, resulting in increased rates of morbidity and mortality. Even more disturbing is that tobacco use is highest among people with the lowest levels of income and education. Thus, reaching these populations is a challenge facing tobacco control programs. This report describes an innovative pilot project implementing a systems change model that involves multiple stakeholders in integrating evidence-based cessation strategies into federal Head Start programs, which serve low-income adults and their children. The Tobacco Cessation Initiative was developed through a partnership between the American Legacy Foundation, the Mailman School of Public Health at Columbia University, and the Louisiana State University Health Sciences Center School of Public Health. The partnership developed guidelines to fit into the overall mission of Head Start by enabling participating sites to incorporate tobacco cessation identification and referral protocols into their existing infrastructures. This program allowed Head Start sites to incorporate, into their existing family services, protocols for user identification and referral; build partnerships with groups supporting tobacco cessation; link families to cessation services; and educate families about risks associated with exposure to secondhand smoke. Applying system strategies in non-clinical settings such as Head Start offers a way to improve the health and quality of life of preschool children at the highest risk for exposure to secondhand smoke.

## Introduction

Although the prevalence of tobacco use is decreasing, one of five adults in the U.S. still smokes, and cigarette smoking continues to be the leading cause of preventable illness and death [1]. The highest rates of tobacco use occur among people with the lowest levels of income and education. Low socioeconomic status (SES) contributes to higher rates of tobacco use and of tobacco-related morbidity and mortality, and, for those in this category, there is limited access to tobacco cessation treatment and prevention [2].

Low SES and tobacco is an issue for children as well as adults, for 26.2 % of children live in households where someone smokes. Moreover, in households below the poverty line, the percentage is 36.9 % [3]. Children in smoking families have substantial exposure to secondhand smoke (SHS), to which there is no risk-free level of exposure [4]. Nearly nine million preschool children are exposed to SHS, a cause of low birth weight, SIDS, asthma, bronchitis, pneumonia, middle ear infection, and other diseases [4].

Although smokers with lower levels of education generally have less knowledge about the negative health effects associated with smoking, many are interested in quitting [5]. Evidence-based treatments and clinical interventions can assist these individuals in quitting [6]. A challenge to tobacco control programs is to make evidence-based cessation strategies available to those in greatest need. In this regard, Head Start (HS) programs have access to low-SES smokers and to the children most vulnerable to SHS exposure.

This paper describes the development of the HS Tobacco Cessation Initiative, a program promoted by The American Legacy Foundation (Legacy), to incorporate into existing services, protocols to engage families in discussions about tobacco use, to identify tobacco users in households, to build partnerships with groups providing cessation services, and to educate families about risks associated with exposure to SHS.

## Methods

This study was approved by the Institutional Review Board of Louisiana State University Health Sciences Center (LSUHSC). The concept for engaging HS sites in tobacco cessation developed from a partnership between Legacy and the Mailman School of Public Health (MSPH) at Columbia University. Free to Grow (FTG), a community-based initiative of the MSPH, provided entry to 15 HS sites. Of these, 4 agreed to participate in the formative phase of the Initiative.

Once the four pilot sites were selected, Legacy and MSPH entered into collaboration with the Behavioral and Community Health Sciences Program of the LSUHSC School of Public Health. The experience of this group in initiating systems change in organizations serving high-risk populations was integral to the development of Initiative guidelines, which were designed to enable participating sites to incorporate cessation identification and referral protocols into their existing infrastructures [7, 8]. This group developed protocols for implementing the Initiative and strategies for incorporating the Initiative into HS programs.

**Fig. 1:**
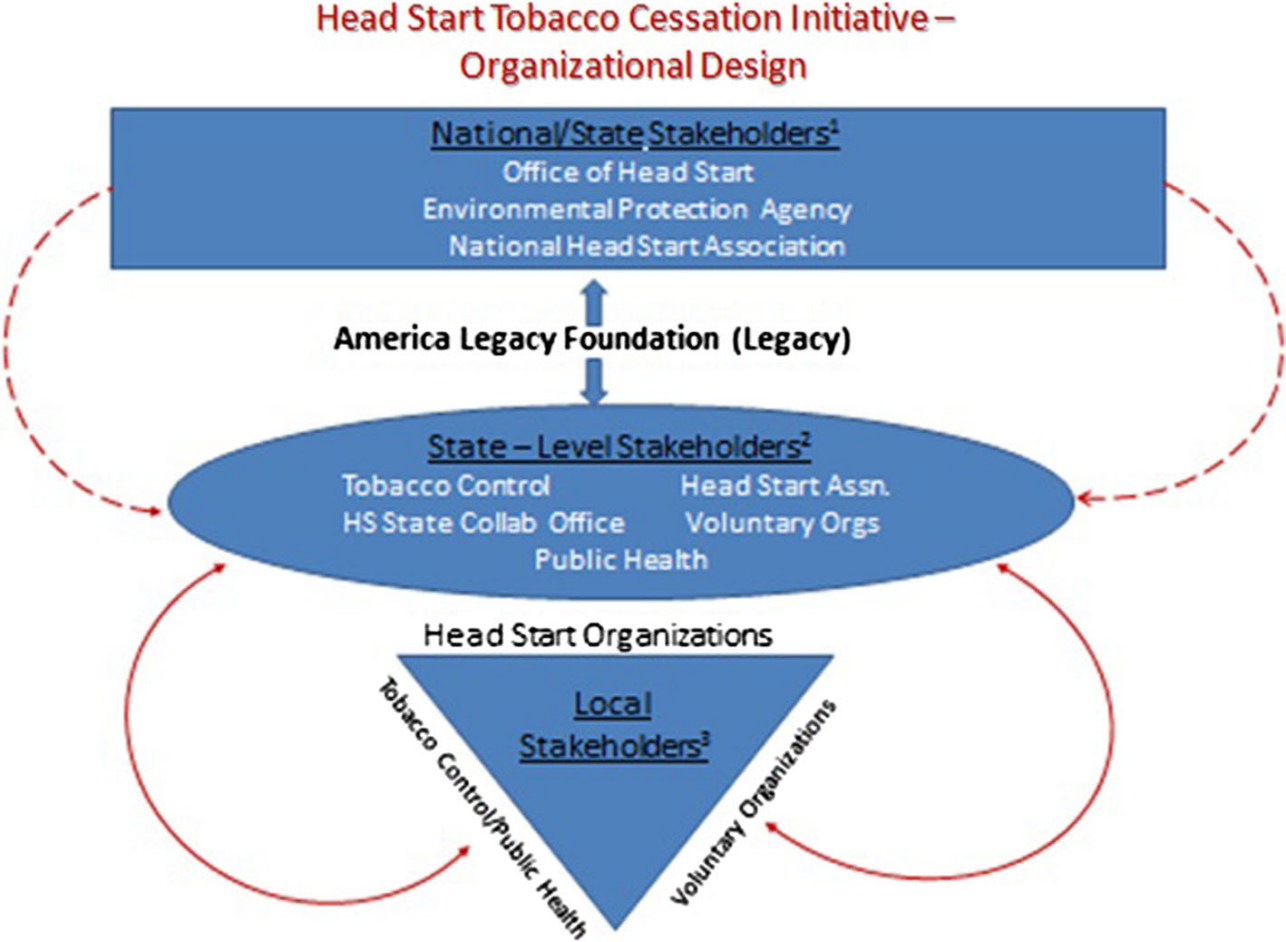
Head Start Tobacco Cessation Initiative—Stakeholders. The role of Legacy in the development of relationships among national, state, and local stakeholders in support of the Head Start Tobacco Cessation Initiative

Figure 1 illustrates the range of relationships developed among stakeholders involved in the Initiative. At the national level, stakeholders included the Office of Head Start, the federal agency responsible for programmatic and fiscal oversight for all HS programs; the National Head Start Association, a membership-based organization representing HS programs and providing advocacy, training, and technical assistance; and the Environmental Protection Agency, a federal agency emphasizing prevention of exposure to SHS. On the state level, stakeholders included State Tobacco Control Programs and agencies responsible for overseeing tobacco cessation and prevention activities, including the provision of services and administration of grant funding to provider organizations; the State Head Start Collaboration Office, where HS staff members facilitated collaboration among HS agencies and state and local entities as charged by the Office of Head Start; Head Start Associations, the voluntary organizations in each state representing the interests of the HS programs at the state and local levels; and State Offices of Public Health, which often house the tobacco control and other maternal and child and chronic disease prevention programs. On the local level, organizations that provide tobacco cessation and prevention services and which were appropriate partners for HS programs were also stakeholders.

## Results

There were three phases to this project: formative pilot, program implementation, and site expansion. The Initiative started in 2004 with a 15-month formative phase supported by Legacy. Four HS sites participated in this phase; these sites are described below. Program development continued in 2006 with an implementation program in Pierce County, Washington, and, in 2007, with a more extensive effort in King County, Washington. The goal of this process was to integrate tobacco control strategies into existing HS infrastructure and protocols and to identify strategies for statewide implementation. Since all HS children and their families receive a screening for needs assessment, adding tobacco use and SHS exposure to HS forms provided a framework for including tobacco control in existing protocols.

The formative pilot (Phase I) was designed to determine the feasibility of applying, in an HS setting, strategies to integrate, into existing practices, identification and treatment of tobacco use as recommended by the U.S. Public Health Service (USPHS) [6] and engagement of tobacco users for referral to local cessation resources. The 15-month pilot program was launched at four HS sites that reflected a range of demographic and geographic characteristics. These were Umatilla–Morrow Head Start (UMHS), in Hermiston, Oregon; Maui Economic Opportunity (MEO), in Wailuku, Hawaii; the Marathon County Child Development Agency (MCCDA) in Wausau, Wisconsin; and the Community Action Project (CAP) in Tulsa, Oklahoma. UMHS has a large Latino population; MEO serves a large Native Hawaiian community; MCCDA supports a growing Hmong population; and the CAP relates to large African American and Hispanic groups. Each of the four sites, which received small seed grants from Legacy for creation and implementation of their model programs, developed procedures that reflected their community context within an overall framework of standardized steps designed for implementation in HS centers nationwide.

During Phase I, a series of surveys was administered to determine the extent to which the four sites focus on addressing tobacco issues with their HS families. HS directors completed a modified Baseline Facility Survey (BFS) used in the public hospital system in Louisiana [7]. The directors of each HS site were asked to report on center practices and policies regarding tobacco use and cessation services, cessation interventions, efforts to monitor tobacco use among parents, budgets for tobacco control activities, tobacco control practices, policies of affiliated health care providers, and strategies and barriers to increase tobacco cessation activities.

The results indicated that directors of the pilot sites recognized the need to change the priority given to addressing tobacco use among HS families; could identify system and resource barriers to tobacco cessation education, training, and outreach; could recommend mechanisms to identify tobacco users; and could educate staff about tobacco cessation and outreach (Table 1).

**Table 1:** Options for implementation of cessation-related activities developed in Head Start formative pilot sites in Oregon, Hawaii, Wisconsin, and Oklahoma, 2004–2006

Cessation activities	Option 1	Option 2	Option 3	Option 4	Option 5
Referral to partner for services	X	X			X
HS provides services (Staff trained in cessation curriculum)			X	X	X
Follow-up by family service workers	X		X	X	X
Quit-line	X	X	X	X	X
Pharmacotherapy	X	X	X	X	X
Support by HS staff (i.e., HS-sponsored support group)		X	X	X	X

In October 2004, 15 people, representing the four sites, attended a 2-day training session. Legacy provided information about nicotine addiction, evidence-based cessation resources, and the principles and practice of Brief Tobacco Intervention (BTI) [9], which is effective in helping low-income individuals quit smoking [10]. Legacy also presented training in motivational interviewing (MI), a counseling approach that engages intrinsic motivation [11].

The participants were then able to (a) identify common opportunities and challenges to incorporating tobacco cessation activities into HS environments; (b) draft an implementation plan for cessation activities for presentation to their site directors; (c) identify target populations for inclusion in the cessation models; and (d) perform a preliminary survey of community cessation resources.

Personnel at each of the four pilot sites developed cessation models that reflected their community context and partnerships with local agencies addressing tobacco issues. Elements associated with program options varied in complexity, intensity, and staff involvement. There were 5 different options for implementation of cessation-related activities (Table 1). Option 1 was limited to identification and referral of household tobacco users to external cessation services and Option 5, the most comprehensive, required HS staff members to include tobacco users on their case loads. Most often, pilot sites adopted Option 2, a model requiring systemic changes for identification and documentation of household smoking, referral to community partners for counseling and pharmacotherapy, and support from HS staff. Table 2 outlines the 13-month sequence of events for approval, preparation, and implementation of the tobacco intervention at HS sites. Following training and organizational assessments, participating sites used their enhanced capacity to address tobacco use among their families.

**Table 2:** Start-up timeline for the Tobacco Cessation Initiative identified in the Head Start formative pilot, 2004–2006

Activities	Months												
	1	2	3	4	5	6	7	8	9	10	11	12	13
Assess interest of stakeholders in the initiative	X	X	X	X	X								
Initiate training						X	X						
Identify cessation resources and partners								X	X	X	X		
Provide additional training, revise protocols, create referral process												X	X
Develop memoranda of understanding with referral organizations													X

The MEO site consisted of 15 centers with 70 staff members serving 298 families. Two HS and eight staff members of partner organizations were trained to provide cessation services. Of the 66 HS households identified as having at least one smoker, 14 individuals accepted a referral, and five of these made a connection with the cessation resource. Tobacco education and cessation information were provided to 300 adults.

The CAP served 1262 families, all of whom were queried about household smokers and interest in cessation. All households with smokers (n = 325) received smoking cessation educational information and referral information. Of these, 45 indicated an interest in attending smoking cessation groups; 7 participated in one or more sessions; and 15 reported a contact with the state quit-line.

UMHS developed a cessation resource guide and informed their staff members about cessation resources in the community. They added cessation education for families to their regular caseload. Parents in selected centers were shown a 5-min video on SHS. In conjunction with completing a questionnaire, 2350 persons who were enrolled in the Women, Infants and Children program received tobacco education. Of the 478 families enrolled in HS centers, 45 had at least one smoker in the household; seven accepted referral to cessation services; and 4 connected with the referral resource.

Marathon County Child Development Agency used a similar approach, informing 50 staff members about community resources and about referring HS families to community-based cessation providers. In HS households, 146 smokers were identified. Of these, 22 were interested in quitting soon; 28 wanted to quit in the next 30–60 days; 16 wanted to quit within the next year; and 80 were not interested in quitting.

The *Phase I* formative pilot provided data on the feasibility of using systems change strategies to integrate the identification and treatment of tobacco use recommended in the USPHS 2000 clinical practice guideline in an HS setting. It identified common points of access, existing practices, and personnel most likely to engage families about tobacco use. These activities identified useful strategies that provided a framework for the next phase of the project, program implementation.

*Phase II*, *Program Implementation*, was conducted in Pierce County, Washington, at seven HS sites. All HS families were educated about the health risks associated with exposure to SHS, and households with tobacco users were assisted with obtaining cessation services. Strategies, identified in Phase I, included staff training, systems change, and the establishment of new partnerships. Staff training, conducted by regional and national personnel, included tobacco education classes, introductions to BTI and MI, discussion of local and state cessation resources, and alteration of HS forms to identify tobacco users. The family assessment allowed discovery of families with tobacco users. Forms were modified to include questions on tobacco use among family members, household rules restricting tobacco use, and children’s exposure to SHS.

In *Phase II*, the timing and processes for integrating tobacco cessation programming into HS facilities were refined. For tobacco programming to occur in tandem with the HS calendar, staff training and system changes were accomplished in the year prior to program initiation. Changing HS forms improved identification of tobacco users and prompted discussions about tobacco use. One finding in this phase was that, in HS, the months of October through February, during which time new and returning students are enrolled and registered, offered the best opportunity to complete training, change forms, and develop new partners.

In 2007, the Initiative was expanded to additional sites as *Phase III* to include Public Health—Seattle & King County (PHSKC) in Seattle, Washington, and 12 HS sites, which included 219 staff members and the capacity for 1,294 child enrollees. This phase focused on expanding the reach of the Initiative and understanding the level of technical assistance and stakeholder involvement needed for implementation. The majority of these sites were served by an agency that also served the *Phase II* sites in King County and had been exposed to form changes through this agency before *Phase III* began. Baseline assessment of new sites was accomplished by interviews with the site directors or their designee between January and March, 2008. For follow-up, the assessment was re-administered one year later. As in Pierce County, the HS staff in King County received training regarding problems associated with use of tobacco, BTI, MI, and local cessation resources. PHSKC also made small reimbursement-based grants of $2,500 available to sites each of 2 years. Sites mainly used funds for materials and food for family activities. Representatives from nine of the sites attended a two-day group training prior to initiation of the effort in December. At all facilities, on-site training was conducted by PHSKC educators in June, 2008. Training ranged from 2 to 6 h. One site received an introductory training on tobacco issues, while all others had training on BTI. Five sites also had MI training on-site. Receptiveness to and attitudes about tobacco intervention were assessed before and after training. Participating in the training sessions were 101 staff members (52 %); representatives from six of the twelve sites completed both pre- and post-training assessments. Assessment of attitudes about SHS exposure and tobacco interventions before and after training indicated that staff entered training believing that counseling families about SHS exposure and tobacco cessation was important. Prior to training, however, few were confident in their skills for treating tobacco use; after training, there was a fourfold increase in staff confidence.

During Phase III, home visits, site-based support, educational groups, and meetings with parents provided opportunities to engage parents about tobacco use. At baseline, both resource and system barriers to addressing tobacco use were identified. These included lack of knowledge and education, competing priorities for resources and time, and the lack of a system for screening and utilizing information on smoking status. Ten sites participated in a 12-month follow up and reported increases in staff training to conduct brief interventions for tobacco use (from 1 to 10 sites), delivery of tobacco interventions (from 4 to 9 for advising, 6 to 10 for referring), systems to document tobacco use and smoke exposure (from 7 to 9), and use of data regarding family tobacco use and SHS exposure to provide resources (from 6 to 10). Nine sites reported working with 109 families to address tobacco use and exposure to SHS, and one site introduced a written plan for addressing tobacco use and SHS exposure. Competing priorities and lack of systems persisted as barriers at follow-up, but lack of knowledge did not.

## Discussion

The HS Tobacco Cessation Initiative was grounded in a systems-change approach designed to increase the capacity of HS centers in addressing high rates of tobacco use among low SES families and the exposure of children to SHS. Strategies included adding questions about tobacco use to HS standard forms; enhancing protocols to assess tobacco use; helping administration and staff understand why tobacco control should be a priority; and training staff in how to engage family members in discussions about tobacco use, SHS exposure risks, and cessation, and to make appropriate referrals to cessation support groups [12]. Protocols and activities associated with the Initiative were incorporated into the routine operations of HS centers and their partners.

In Phase I, three steps were identified as essential for implementation of cessation support within the HS structure: (1) training to increase staff knowledge of the effects of tobacco use and skill building in the use of MI and BTI; (2) establishing relationships with local and state tobacco cessation providers; and (3) revising HS protocols to identify, engage, and refer family members who use tobacco to appropriate cessation services.

Phase II confirmed the following: (1) County health departments will support HS and provide resources to engage families about tobacco use and SHS exposure and provide technical assistance to ensure implementation of newly acquired skills, such as MI and BTI. (2) Existing assessment protocols and family support practices within HS programs provide a point of access for identifying tobacco users. Embedding tobacco questions in the HS forms allows discussions about tobacco use and permits staff to explore readiness to make a quit attempt and willingness to ban or restrict household tobacco use. (3) Modifying forms and program practices should begin 6–9 months prior to the start of a school year and should include input from governing entities and site personnel.

In Phase III, resource and system barriers to addressing tobacco use were a key focus, and staff training was identified as an essential component of the Initiative. Follow-up assessment found changes in practices to deliver tobacco interventions, including systematic changes in documentation.

Previous studies indicate that after participation in an MI counseling session, parents smoked fewer cigarettes and household nicotine levels decreased [13, 14]. In this Initiative, MI was identified as an important skill useful to HS staff for engaging families about tobacco use.

The systems changes observed in this project required the support of administrators and others responsible for shaping programmatic focus and for building staff support and capacity. Program directors participated in discussions about the importance of addressing tobacco use among HS families, training staff to engage families about tobacco use, and including tobacco assessment and engagement as a component of staff supervision. Each was considered a catalyst for adoption of the Initiative, and for improving the acceptability of engaging families about tobacco use as a part of the commitment to the well-being of the enrolled children. Implementation of the Initiative required formation of relationships between HS staff and local health departments, cessation providers, state health departments, and state quit-line providers. As such, system changes will be more likely achieved when the National Office of Head Start adopts performance standards that specifically address tobacco use and SHS exposure.

Limitations: while aspects of systems change could be directly observed (e.g., changes to forms), changes in practice (e.g., increased advising) were measured by report of site directors or designees. Direct measurement of these changes and the extent to which they are sustained is warranted.

To achieve widespread changes in HS staff and family behaviors, implementation must focus building a sustainable delivery model that includes training personnel to interact with the priority population and expanding strategically across HS sites [15]. Applying system strategies in non-clinical settings such as HS offers a way to improve health and quality of life of preschool children at risk for exposure to SHS. Nationwide application will require broadening the cadre of stakeholders so that national policy can shape practice, and local practices can be supported by state and local entities promoting tobacco control.
